# Effect of sublethal concentrations of glyphosate-based herbicides (Roundup Active®) on skin of the tropical frog (*Dendropsophus molitor*)

**DOI:** 10.1007/s11356-023-29816-8

**Published:** 2023-09-30

**Authors:** Catalina López-Flórez, Monica Andrea Ortíz Ruíz, Edwin Gómez-Ramírez

**Affiliations:** Faculty of Applied Basic Sciences, Universidad Militar Nueva Granada, Cajicá, Km 3, Cajica, Colombia

**Keywords:** Herbicide, Tadpole, Amphibians, Chronic toxicity

## Abstract

In Colombia, glyphosate (GP) is used to control weeds, with Roundup Active® being the most widely used. This use has affected aquatic ecosystems, causing malformations in amphibians. The Savannah frog (*D. molitor*) is a tropical frog inhabiting the mountain of Colombia. In the present study, we determined the effect of sublethal concentrations of GP (Roundup Active®) on the skin of *D. molitor*. Twenty-four tadpoles were exposed to concentrations of GP (T1: 0, T2: 1.4, T3: 3.6, and T4: 5.6 a.e mg/L) during 31 days. In 10 individuals per treatment, two skin regions were evaluated: dorsal cranial and caudal ventral to determine histopathological alterations. Morphometric analysis of the layers of the skin was performed: epidermis, dermis, and hypodermis-muscular. T1 did not present histopathological alterations. Since T2 was identified, glandular cell hyperplasia and hypertrophy increased melanophores and melanin accumulations in the highest concentrations of GP. The ultrastructure revealed an increase in excretory glands in the dermis. In the other layers, an increase of melanophores and melanocyte clusters was observed accompanied by vacuolization of basal cells. The morphometry showed an increase in the thickness of the dermis in the dorso-cranial region in T2 compared to the other treatments, while the ventral caudal region exhibited a variation in the thickness of the dermis from T2 and a decrease in T4. Despite evaluating sublethal concentrations, the skin of *D. molitor* tadpoles presents histopathological, ultrastructural, and morphometric alterations that could affect the survival of the species in the natural environment.

## Introduction

GP (N-phosphonomethyl glycine) is a broad-spectrum, non-selective, systemic herbicide used to control annual and perennial plants (Peillex and Pelletier 2020). GP inhibits the synthesis of aromatic amino acids such as tryptophan, phenylalanine, and tyrosine by suppressing the enzyme 5-enolpyruvylshikimate-3-phosphate synthase (EPSPS) of the shikimate pathway, affecting plant development (Zulet-González et al. 2020). As expected, it did not affect animals, since they lacked this metabolic pathway (Cavalier-Smith 2007).

In Colombia, GP is used to control weeds in agriculture from 76 to 90% and for the eradication of illicit crops from 10 to 14% (Solomon et al. 2007), which has represented a greater environmental risk for amphibians due to the contamination of terrestrial and aquatic ecosystems (Sasal et al. 2017). The commercial presentation of GP most used in Colombia and the world is Roundup®, which contains GP and a surfactant, mainly polyoxyethylamine (POEA), which has become more toxic in amphibians (Georgieva et al. 2018), fish (Eslava-Mocha et al. al., 2007), reptiles (Wagner et al. 2013), and mammals (Ackermann et al. 2015). Additionally, this combination present in several glyphosate-based herbicides can be more toxic in aquatic ecosystems by interfering with the skin and gill respiration of tadpoles and fish (Eslava-Mocha et al. 2007; Georgieva et al. 2018).

Amphibians are an endangered group with the highest rates of species extinction, due to several factors such as habitat loss, environmental pollution, climate change, and different fungal and parasitic diseases (IUCN 2013; Deknock 2020). This group plays a fundamental role in pest control of different crops, so they are continuously exposed to agrochemicals, making them excellent study models for the post-exposure effects of different xenobiotics or pollutants (Peltzer et al. 2010; Svartz et al. 2012; Turani et al. 2019). The toxicological interest in amphibians is even more than in other organisms due to the unique characteristics of their epidermis and the functions that are related to it, such as cutaneous respiration, avoidance of water and ion loss, and protection against biological and/or chemical contaminants (Gordon et al. 2015; Kaufmann and Dohmen 2016).

**Fig. 1:**
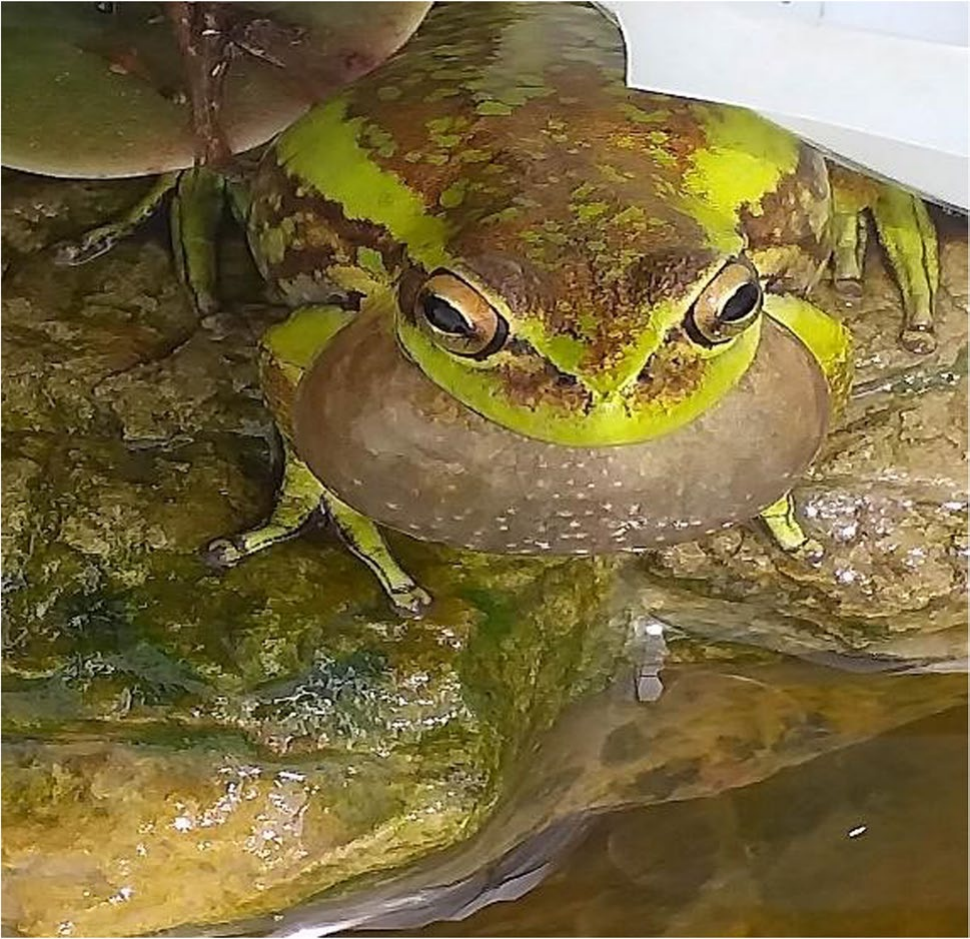
*Dendropsophus molitor*. Cajicá, Colombia

The Savannah frog (*D. molitor*) (Fig. 1), lives in the Eastern mountain range of Colombia between 2000 and 3600 m above sea level (Guarnizo et al. 2012), specifically in the Andean region of the country whose main economic activity is agriculture, which depends on the constant use of herbicides such as GP to maintain adequate production (Mink et al. 2011). For this reason, it has been evidenced that the probability of exposure of amphibians to different xenobiotics has increased and is critical for species that breed in small water bodies such as *D. molitor* (Higuera-Rojas and Carvajal-Cogollo 2021).

Previous studies have found that amphibian embryos are more sensitive to environmental toxins than their terrestrial or adult counterparts to be more closely related to water sources for their development, (Üveges et al. 2017; Ujszegi et al. 2021). Despite its importance, neither histopathological nor ultrastructural analyses have been performed on the integumentary tissue of tadpoles exposed to sublethal concentrations of GP, which are more likely to be found in the natural environment. Therefore, the objective of this study was to determine the histopathological and morphometric alterations on the skin of tadpoles of a tropical frog (*D. molitor*) exposed to sublethal concentrations of a commercial presentation of GP.

## Materials and methods

### Biological material

This work was carried out at the Animal Physiology and Embryology Laboratory at the Universidad Militar Nueva Granada in Cajicá, Colombia, (4°56′37″ N–74°00′35″ W, 2560 m.a.s.l.) with annual temperatures between 12 and 19 °C and relative humidity of 88%. Eighty tadpoles of *D. molitor* were captured from the reservoirs under permit Res No. 1198 granted by the Colombian Ministry of Environment. Specimens were acclimatized for 1 week according to studies on other tropical amphibians (Riaño et al. 2020). Tadpoles were at Gosner’s developmental stage 24 (Gosner 1960) and were fed with commercial food Tetra Color Type® (47.5% crude protein) adjusted to 2% of biomass. The water conditions were maintained at pH 7.2, temperature 16 °C, total ammonia nitrogen (TAN) <0.5 mg/L, nitrite <0.5 mg/L, and dissolved oxygen (DO) >5 mg/L.

### Chemicals and experimental design

Four treatments with two replications (4 × 2) were evaluated: T1: 0 mg/L, T2: 1.4 mg/L, T3: 3.6 mg/L, and T4: 5.6 mg/L acid equivalent (a.e)/L present in the commercial product Roundup Active® (Bayer Crop Science). The composition of this commercial formulation is 363 g/L acid GP equivalent to 446 g/L potassium salt of *N*-phosphonomethyl glycine and a surfactant that is not specified.

A total of 10 individuals were analyzed per treatment (*N* = 80). The organisms were maintained in semi-static systems with a total volume of 4 L of tap water per experimental unit for 40 days. Water quality parameters were measured daily: temperature, pH, dissolved oxygen with a HI-9829 Hanna® multiparameter probe, TAN, and nitrite with the Spectroquant Multy® and Merck® high-sensitivity kit. The tadpoles were anesthetized and sacrificed with benzocaine at 0.5 mg/L, following the protocols of Bioethics and animal experimentation exposed by Underwood and Anthony (2020). Then, the samples of integumentary tissue were dissected at different regions: dorsal-cranial and tail in the lateral profile (500 μm^2^). These regions were selected due to the physiological and ecological importance they play in the organism, the integumentary tissue of the dorsal-cranial region protects the brain, and the ventral dorsal region allows a protective or camouflage response to predators and contaminants (Barbosa et al. 2018).

### Sample processing for histological, ultrastructural, and image analysis

The tissues were fixed in 2.5% glutaraldehyde for 2 days, then washed with phosphate-buffered saline (PBS) and post-fixed with 2% osmium tetroxide (Riaño et al. 2020). Dehydration processes were carried out with increasing concentrations of ethanol for 10 min (50%, 70%, 90%, 100%) and pure acetone. The samples were embedded with homogeneous mixtures of Poly/Bed 812® resin and acetone in proportions of 1:2, 1:1, and 2:1, respectively, for 15 min. Finally, the samples were embedded in pure resin for 1 h and 30 min, and polymerized for 24 h at 70 °C in a Thermo Scientific™ incubator (Gómez-Ramírez et al. 2016).

Transverse regions 1 μm thick were made with a Slee Cut 4060® rotary microtome and stained with toluidine blue. The regions were observed and photographed with a high-resolution optical microscope (HROM) ZEISS® equipped with an Axiocam digital camera (ZEISS®). The area of the integumentary tissue layers in each of the treatments (epidermis, dermis, and hypodermal-muscular layer) in the two regions evaluated dorso-cranial and ventral-caudal was determined using ImageJ 1.48v software (https://imagej.nih.gov/ij/, 2013) (Gomez 2016). Six slices per individual were processed with 50 μm separated between them. For ultrastructure analysis, 130 nm slices were made using ultramicrotome Leica® EM UC6. The regions were contrasted with lead citrate/uranyl acetate. The samples were analyzed in an electron microscope of transmission JEOL® JEM-1400Plus 120 kV. Photographs were carried out with a GATAN® camera attached to this equipment and the GATAN® DigitalMicrograph 1.80.70 program.

### Statistical analysis

Data of the thickness of the integumentary tissue layers (epidermis, dermis, and hypodermis-muscle) were evaluated for the assumption of normality by means of the Shapiro-Wilk test, a one-way ANOVA, and the Tukey-Kramer test (*P* ≤ 0.05 type I error) were performed. All statistical analyses were performed using R 3.5.0 Software. 

## Results

**Fig. 2:**
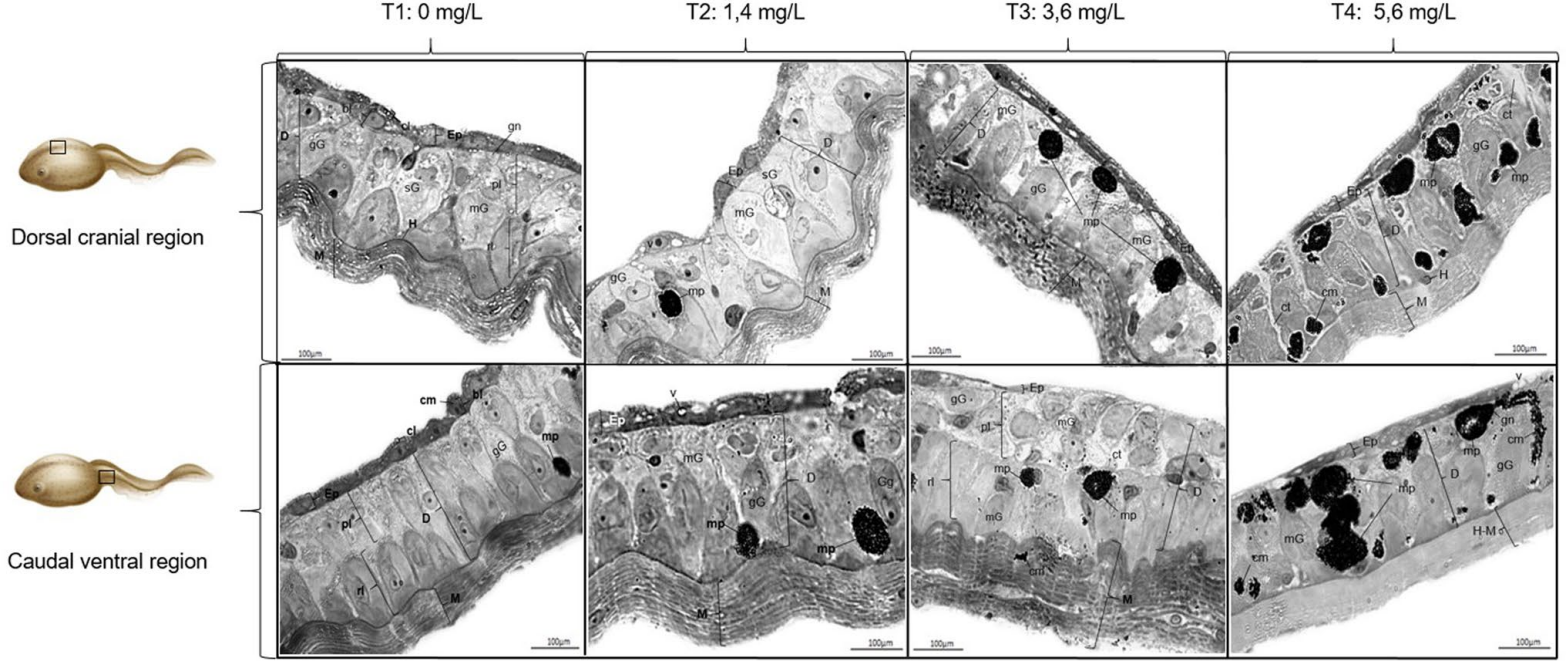
*D. molitor* skin in HROM. Epidermis (Ep), corneous layer (cl), basal layer (bl), dermis (D), papillary layer (pl), reticular layer (rl), hypodermis (H), muscle tissue (M), multinucleated cells (mc), melanophores (mp), melanin clusters (cm), mucous glands (mG), serous glands (sG), granular glands (Gg), glandular neck (gn), connective tissue (ct), vacuolization (v). Toluidine blue stain

**Fig. 3:**
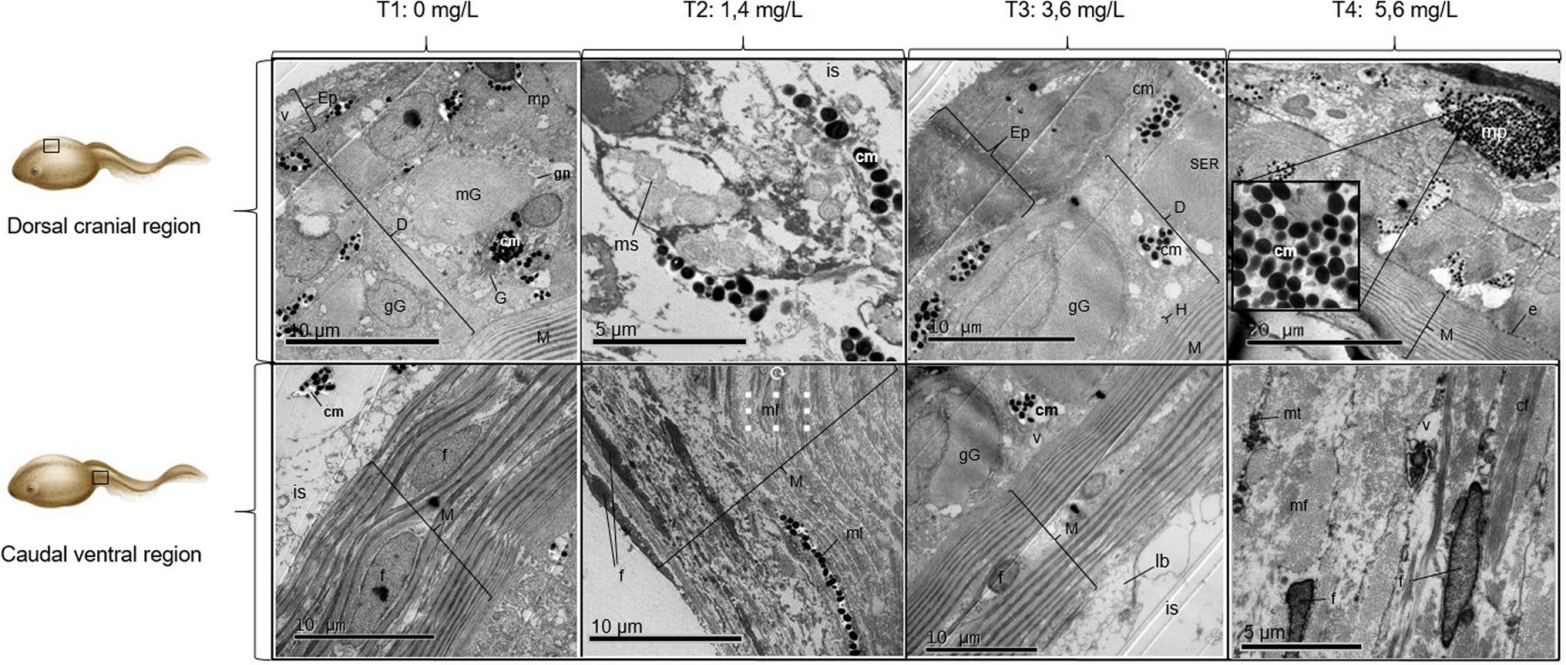
*D. molitor* skin in MET. Epidermis (Ep), dermis (D), hypodermis (H), muscle tissue (M), muscle fibers (mf), fibroblasts (f), lipid bodies (lb), melanin layer (ml), melanophores (mp), immature melanocytes (im), clusters of melanin (cm), endothelium (e), mitochondria (mt), smooth endoplasmic reticulum (SER) associated with granules, Golgi apparatus (G) characterized by flattened cisternae forming dictyosomes, interstitial space (is), multivesicular bodies (mv), mucous glands (mG), granular gland (gG), glandular neck (gn). Toluidine blue stain

**Fig. 4:**
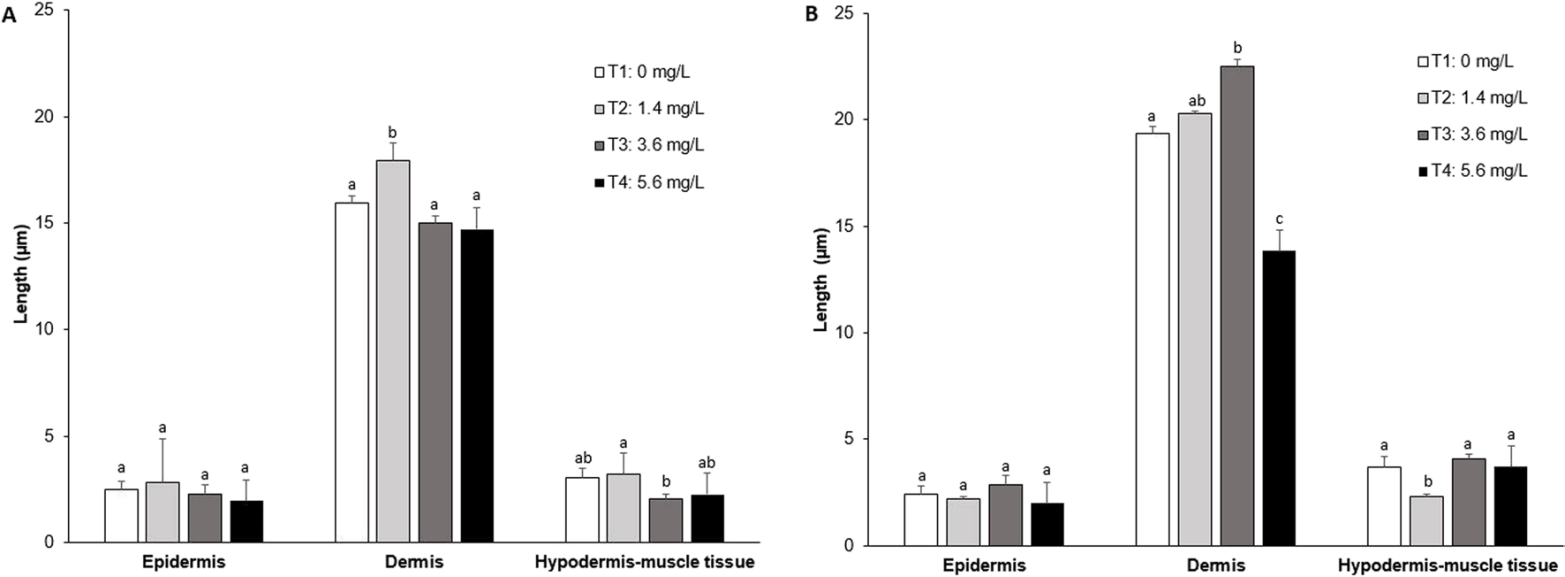
Tegumentary tissue thickness of the dorsal-cranial and caudal ventral region of *D. molitor* exposed to different concentrations of Roundup Active®. Data are expressed as mean ± SD. Different letters represent significant differences (*p* < 0.05)

In HROM and TEM, the integumentary tissue of *D. molitor* was characterized by epidermis, dermis, hypodermis, and muscle tissue. The epidermis is constituted by stratified plane epithelial tissue; this layer is composed of different strata of which the stratum corneum is formed by anucleated flat cells, and the stratum basale which is formed by a layer of cubic cells in differentiation. It was common to observe two nucleoli. The dermis was subdivided into two layers, the papillary layer and the reticular layer. These layers exhibit a loose connective tissue and multicellular exocrine glands composed of mucous and granular cells and a compact stratum with dense connective tissue where melanophores are distributed. In the dermis of *D. molitor*, the same glandular types found in the epidermis were identified although the glands were more abundant. Hyperplasia and hypertrophy of glandular cells were observed especially in T2 and T3 treatments. In the T3 and T4 treatments, an increase in melanin and melanophore clusters was evidenced, which were located in the dermis strata. The hypodermis was characterized by the deepest and thinnest layer of the skin, being closely related to the skeletal muscle tissue (Figs. 2, 3, and 4).

Significant differences were found in the length of epidermis, dermis, and hypodermis-muscle tissue between the dorsal cranial and caudal ventral regions. The epidermal and dermal thickness analyzed in the dorsal cranial region of *D. molitor* increased in T2 treatment in contrast to T1 treatment, whereas the epidermal and dermal thickness analyzed in the ventral caudal region of *D. molitor* increased in T2 and T3 treatment.

## Discussion

### Histopathological effects

The severity of the tissue lesion was evaluated through histological observation and the ultrastructure of the integumentary tissue, finding that the skin in *D. molitor* presented three constitutive layers (epidermis, dermis, and hypodermis) similar to those reported in other amphibian species (Azevedo et al. 2005; Felsenburgh et al. 2007). In the control treatment, T1 did not exhibit histopathological alterations and the cells of each of the layers were normal. In the ventral caudal region of treatments T2 and T3, hypertrophy and hyperplasia were evidenced in the dermis; it is an alteration described in other amphibians as a response to pathogens and the use of pesticides in extensive agriculture that contaminates water bodies (Magnus and Rannap 2019). This fact was similar in the frog *Scinax nasicus* where alterations characterized by cranial and oral deformity and hyperplasia in the epidermis were presented when the organisms were exposed to different concentrations of pure GP (3.07, 3.85, 4.8, 6, and 7.5 mg/L) at 24, 48, 72, and 96 h (Lajmanovich et al. 2003). Layered hypertrophy of the integumentary tissue can cause marked alterations in the shape and structure of the organ; it has been shown that hypertrophy in the epidermis and dermis generates cellular disorders in the connective, epithelial, and glandular tissues, which negatively affects amphibians by reducing cell division (Sahin et al. 2014; Fernández et al. 2017). If these epithelial damages persist in response to pesticides present in the environment, abnormal behaviors and irreversible alterations may occur causing high mortality (Menéndez-Helman 2020).

The dermis was the thickest layer of the integumentary tissue of *D. molitor*; this may be due to the fact that mucous and serous exocrine glandular cells are associated with this layer, which coincides with the study carried out by Toledo and Jared (1995), which showed that these glands are large in response to the synthesis and storage of multiple substances. Therefore, the hypertrophy and hyperplasia in the dermis and, thus, in the papillary and reticular layer, would explain the increase in thickness in the ventral caudal region of *D. molitor*. In treatments, T3 and T4 cellular scalloping is formed in the hypodermis and in the muscle tissue, probably caused by the cytotoxicity of the GP, the surfactants, or the synergistic effect of both substances present in the commercial products of these herbicides, increasing damage to the epidermis and hypodermis by causing structural changes in the membrane of epithelial cells (Monroy et al. 2005; Ramírez et al. 2009). The results obtained in *D. molitor* confirm that the mucous glands are present in both regions, dorso-cranial and ventral-caudal, being more abundant than serous or granular glands, which is related to the protective role of mucus (Sever and Siegel 2015). In the dermis of *D. molitor*, a greater number of mucous than serous glands was observed, which is normal in non-poisonous amphibians because these glands function as a water reservoir, and their different secretions of glycoproteins and polysaccharides prevent mechanical damage and desiccation and partially isolate the organism from its environment (Azevedo 2005; Wells 2007). In treatments T2, T3, and T4, the number of mucous glands increased considerably, these glands are related to respiration and osmotic and water balance in amphibians and, therefore, an increase in the number may be due to the fact that the GP and the surfactant present in Roundup Active® generated a response to stress and there was the greater secretion of mucus as a strategy to isolate the organism from the xenobiotic.

The results coincide with that obtained by Henao-Muñoz et al. (2015), where amphibian species such as *Rhinella humboldti* and *Rhinella marina*, exhibited greater mucus secretion and high mortality when exposed to concentrations of Roundup Active® and Cosmo-Flux® 411F (106.25; 212.5; 425; 850 and 1700 mg/L). On the other hand, the slight increase of the granular glands in T3 and T4 may be due to the that these glands secrete peptides, steroids, and alkaloids under stress conditions such as predation or anthropogenic contamination (Naya et al. 2004).

Physiologically, the increase in mucous glands is a consequence of increasing agrochemical concentrations to enhance mucous secretion and control the skin pH to maintain an osmotic balance and prevent dehydration (Mobarak et al. 2021). Studies suggested that mucus cell hyperplasia reduces xenobiotic diffusion across the epithelium and may be a response to a possible electrolyte imbalance caused by tissue injury (Lin et al. 1995; Ramírez et al. 2009).

The dark pigments of different sizes coincide with histological descriptions of melanin, which are produced by melanophores generally in the compact stratum of the dermis of amphibians (D’Errico et al. 2018). Experimental studies in fish and amphibians indicate that exposure to different doses of agrochemicals based on GP causes integumentary alterations such as hyperplasia and hypertrophy of flat cells of the epidermis, epidermal leukocyte infiltration, and subepidermal accumulation of pigments (Ramírez et al. 2009). This study shows an increase in the number of melanophores and clusters of melanin in the dermis of *D. molitor* in treatments T3 and T4 as a possible response to GP and the surfactant present in Roundup Active®. This alteration is similar to the results described by Kalashnikova (2000) who suggests that the melanin in frogs may be produced by the destruction of erythrocytes (Kordylewski 1984). The increase in melanocytes and melanin is a normal process in aquatic organisms because it is an antimicrobial agent and plays a protective role by reducing the entry of chemical substances into the integumentary tissue (McNamara et al. 2021). In addition, melanin is used in response to factors that may affect the shape and structure of the integumentary tissue of these organisms. The main function of melanin is to protect the individual from environmental conditions that may be adverse, such as ultraviolet radiation and anthropogenic pollution (Cao et al. 2021).

### Ultrastructural effects

Different effects have been reported after the exposure of amphibians to chemical contaminants, such as increased sodium absorption and rapid percutaneous absorption, which may cause a change in the thickness of the integumentary tissue layers (Quaranta et al. 2009).

In the epidermis and dermis, it was common to find vacuolation as hyaline drops in the cytoplasm of the basal cells. This cytoplasmic vacuolation could be a non-specific sign of tissue alteration and a mechanism of the organism to reduce the harmful effect of xenobiotic (Cooley et al. 2000; Sarkar et al. 2005; Caramello et al. 2018). Moreover, the presence of mitochondria exhibiting a matrix with different electron densities and the presence of electron-dense granules in the dermis, hypodermis, and muscle tissue suggests that the Roundup Active® promoted physiological alterations in the organism as a mechanism to repair cellular damage in adaptation to the contaminated environment (Strilbyska et al. 2022).

An increase in the thickness of the dermis was observed due to excretory structures, while in some amphibian species these structures are related to serous glands; in *D. molitor*, it was possible to observe three types of glands; serous, mucous, and granular, the last two characterized by the condensation of some granules without substructures and with a major electron-dense content (Brunetti et al. 2012). The melanocytes were identified by the presence of numerous electron-dense melanin granules, associated with melanophores that presented cytoplasmic expansions with dispersed melanosomes. The melanophores in amphibians are found in the papillary or reticular layer of the dermis and exhibit prolongations to superficial dermal levels related to the basal cell layer of the epidermis (Narins et al. 2001). The treatments T2, T3, and T4 showed muscle atrophy, decreased thickness of fibroblasts, scattered and necrotic muscle fibers, and granular cell infiltration. This may be due to the fact that the compounds present in Roundup Active® cause progressive muscle degeneration by altering various biochemical processes, affecting cell membranes and various organelles (Castañé et al. 2003).

### Morphometry

Morphometric analysis showed significant differences in the thickness of the layers composing the integumentary tissue in *D. molitor*. The thickness of the integumentary tissue and its component layers varies in anurans according to the body region analyzed (Toledo and Jared 1995). *Leptodactylus* species of the fuscus group exhibit differences in the integumentary tissue thickness as a result of cell differentiation and morphological changes in the epidermal cells (García et al. 2011).

The thickness of the dermis in the dorso-cranial section of *D. molitor* decreased compared to the dermis in the caudal ventral region. This could be due to the fact that mucous glands are produced in this layer and decreasing their thickness allows mucus to reach the epidermis in order to isolate the organism from the toxic substance or to act as an expulsion mechanism of the xenobiotic (Mosley et al. 2018). The increased production of mucus in the gills and skin is a response described in fish exposed to environments contaminated with GP-based herbicides, where this mucus improves the removal, dilution, and/or neutralization of toxic and pathogenic compounds, reducing the diffusion of harmful agents into the blood (Speare and Ferguson 2006). It is known that the exposure of different organisms to chemical products can cause alterations in tissues and organs such as the skin (Hayes et al. 2010).

Studies show an increase in cutaneous parasites in *Lithobates pipiens* exposed to a combination of the pesticide Atrazine with phosphate, causing immunosuppression and impairment in tadpoles and adults of this species (Gavel et al. 2021). Lysis in epithelial cells has also been reported in different organs of aquatic organisms such as amphibians exposed to surfactants; hence, the differences found in the integument of amphibians are related to the levels of human disturbance and pollution to which the organisms are exposed (Rohr et al. 2008; Bach et al. 2018). It was observed that the thickness of the dermis was greater compared to the other layers evaluated. The results coincided with that described by García et al. (2011), who reported that the thickness of the dermis is greater in areas of the skin that contain glands than in those that do not, in order to provide metabolic support to the glandular tissue in this integumentary layer.

On the other hand, Bach et al. (2018) found deleterious effects of GP in *Leptodactylus latrans* tadpoles exposed to a commercial presentation of GP Roundup UltraMAX®, finding a significant increase in the number of melanomacrophagic cells and melanomacrophagic centers in the liver, as well as histological alterations associated with lipidosis and hepatic congestion. Also, Soloneski et al. (2016) determined acute toxicity of the mixture of Banvel® based on dicamba (DIC) and Credit® based on GP, demonstrating a synergistic effect of the mixture of GP and DIC in inducing DNA damage of blood cells in late-stage *Rhinella arenarum* larvae. Another study with *Rhinella. arenarum* carried out by Lajmanovich et al. (2003) confirms that the exposure of this species to organophosphate pesticides such as 2,4-D, chlorpyrifos, and GP generates neurotoxicity, oxidative stress, and immunological depression. Howe et al. (2004) compared the acute toxicity of Roundup Original®, and the surfactant polyoxyethylamine (POEA) present in Roundup Transorb® formulation to four North American amphibian species (*Rana clamitans*, *R. pipiens*, *R. sylvatica*, and *Bufo americanus*) obtaining a decrease in the snout-cloacal length, an increased time to complete the metamorphosis, tail damage, and gonadal abnormalities due to the interruption of hormonal signaling. This is the first study of the effects of sublethal concentrations of a GP presentation on the skin of the tropical tadpole, *D. molitor*. The alterations found in *D. molitor* could affect embryonic development and thereby reduce the body size of individuals, and their swimming ability prevents the search for food and escape from predators, and finally can cause the death of the individual, impacting the population size of this endemic species (Kats et al. 2000; Ezemonye and Ilechie 2007; Relyea 2012).

## Conclusions

The main histopathological alterations found in the integumentary tissue of *D. molitor* were glandular cell hyperplasia and hypertrophy, increased melanophores, and melanin accumulations in the highest concentrations of GP present in Roundup Active® herbicide. Regarding ultrastructure, a considerable increase of excretory glands in the dermis was observed. In the layers evaluated, epidermis, dermis, hypodermis, and muscular tissue, an increase of melanophores and melanocyte clusters with melanin granules was observed, accompanied by vacuolization of the basal cells, while for the dermis and hypodermis, muscular tissue exhibited an increase in electron-dense material, muscular atrophy, and a decrease in fibroblasts as a response of the organism to the xenobiotic. The morphometric study showed a decrease in the thickness of the dermis in the dorsal cranial region in contrast to the ventral caudal region, possibly to allow greater availability of mucus on the epidermal surface, thereby hindering the entry of the xenobiotic into the organism.

## Data Availability

The datasets used and analyzed during the current survey are available from the corresponding author on reasonable request.
